# A Peculiar Plant

**Published:** 1898-02

**Authors:** 


### A Peculiar Plant.

A plant grows in Assam, the botanical name of which is Gymnema sylvestre, and which has the peculiar property, when chewed, of temporarily neutralizing the sense of taste as regards sweet and bitter things, while sour and saline substances remain unaltered. The Hindus claim that the plant is an antidote to snake bite. However that may be, it is believed that the plant might be advantageously introduced in our pharmacopœia as a means of disguising the bitterness of quinine and other disagreeable medicines.

